# Dizziness and slow heart rate during exercise

**DOI:** 10.1007/s12471-017-0985-0

**Published:** 2017-04-11

**Authors:** R. Joustra, M. Boulaksil, H. W. Meijburg, J. L. Smeets

**Affiliations:** 1 Department of Cardiology, Jeroen Bosch Hospital, ’s-Hertogenbosch, The Netherlands; 2 Department of Cardiology, Radboud University Medical Center, Nijmegen, The Netherlands

## Answer

The ECG at presentation shows an irregular baseline and no *P*-waves, suggesting atrial fibrillation. A regular multiform ventricular bigeminy with fixed coupling interval can be appreciated, which could be typically seen in digitalis intoxication [1].

Other potential ECG features of digitalis intoxication include decreased AV conduction (because of increased vagal effects on the AV node), possibly resulting in high grade AV block, and enhanced automaticity with frequent premature ventricular complexes. Enhanced automaticity may also induce bidirectional tachycardia with alternating left and right bundle branch block pattern, which is most commonly associated with digitalis toxicity [2].

In our patient, the first complex of the ventricular bigeminy has a left bundle branch block pattern with steep initial ventricular activation, suggesting a focus originating in or near the specific conduction system. The second complex of the bigeminy has an intermediate electrical axis, a left bundle branch block-like morphology, tall R‑waves in the inferior leads and a negative QRS-complex in aVL, suggesting a right ventricular outflow tract focus. Although coupling intervals of the bigeminal complexes are fixed, not all RR intervals are at equal length. Therefore, a bidirectional tachycardia is excluded and renders a multifocal origin of the ectopic ventricular complexes most likely.

**Fig. 1:**
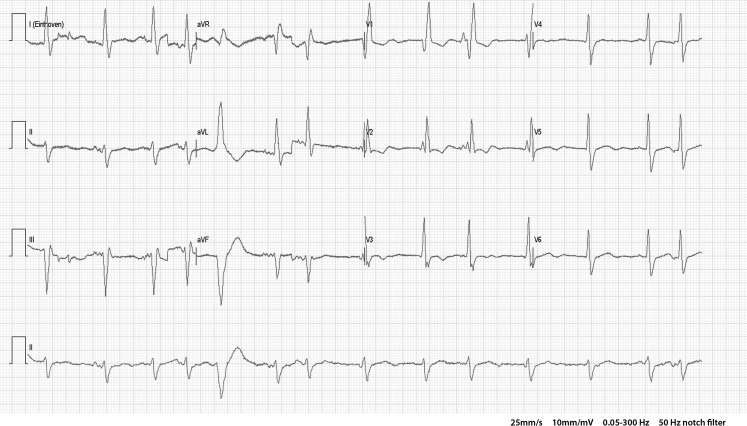
ECG one month before presentation

A previous ECG (Fig. 1) showed atrial fibrillation and a right bundle branch block. This finding and the fact that no retrograde *P*-waves are seen in between the bigeminal QRS-complexes rules out escape-capture bigeminy as an alternative explanation and favours impulse formation in or near the specific conduction system.

Although our patient had been treated with the lowest daily dose of digoxin (0.0625 mg q.d.), she nevertheless showed clear signs of digoxin intoxication. This may have been driven by progressive renal failure. Considering the known overlap of serum digoxin levels between groups with and without toxicity [3], we decided not to measure serum levels of digoxin. In addition, no digoxin-specific Fab antibodies were administered since our patient remained haemodynamically stable and she did not develop any other life-threatening complications such as ventricular tachyarrhythmias or bradyarrhythmias.

We decided to implant a two-chamber pacemaker and subsequently reinitiate the beta blocker aiming to optimise rate control, as she developed atrial arrhythmias interspersed with sinus bradycardia (brady-tachy syndrome) one week after discontinuation of digoxin and bisoprolol. Thereafter, she recovered well and was discharged three days after implantation.

Conclusion: multiform ventricular bigeminy with fixed coupling interval caused by digoxin intoxication.
